# Precontractile optical response during excitation-contraction in human muscle revealed by non-invasive high-speed spatiotemporal NIR measurement

**DOI:** 10.1038/s41598-017-18455-y

**Published:** 2018-01-09

**Authors:** Markus Lindkvist, Gabriel Granåsen, Christer Grönlund

**Affiliations:** Department of Radiation Sciences, Biomedical Engineering, Umeå University, Umeå, 90187 Sweden

## Abstract

During muscle contraction the excitation-contraction process mediates the neural input and mechanical output. Proper muscle function and body locomotion depends on the status of the elements in the same process. However, non-invasive and *in-vivo* methods to study this are not available. Here we show the existence of an optical response occurring during the excitation-contraction process in human biceps brachii muscle. We developed a non-invasive instrument from a photodiode array and light emitting diodes to detect spatially propagating (~5 m/s) and precontractile (~6 ms onset) optical signals closely related to the action potential during electrostimulation. Although this phenomenon was observed 60 years ago on isolated frog muscle cells in the lab, it has not been shown *in-vivo* before now. We anticipate our results to be a starting point for a new category *in-vivo* studies, characterising alterations in the excitation-contraction process in patients with neuromuscular disease and to monitor effects of therapy.

## Introduction

Proper muscle function and body locomotion relies on the excitation-contraction (EC) process, which mediates the neural input and mechanical output of the muscle. However, non-invasive and *in-vivo* methods to study EC are not available.

Optical techniques utilising near infrared (NIR) light have enabled several important advances in the study of brain and muscle tissues^1,2^. The use of wavelengths between 600 and 900 nm allows penetration depths up to several centimetres, suitable for *in-vivo* and diagnostic applications. Most *in-vivo* and diagnostic applications are limited to assessing slow (~seconds) biological processes such as blood oxygenation. By contrast, 60 years ago optical techniques were used to study transient processes in isolated frog muscles. These *ex-vivo* experiments showed that electrostimulated contraction induces a rapid (~milliseconds) change in optical opacity (we will call it optical response), related to the precontractile period^3–6^, i.e. the time window preceding actual muscle contraction. Recently there has been a growing interest in investigating more rapid processes in the human peripheral nervous system^7^ and muscles. Although NIR methods have been used in several *in-vivo* studies on electrostimulated skeletal muscle tissue^8–10^, no evidence of this precontractile optical response has been put forth in human *in-vivo* non-invasive experiments.

In muscle contraction, the excitation-contraction process is executed in the precontractile period and involves action of the triad structure, comprising the T-tubuli (invaginations of the cell’s membrane) and the sarcoplasmic reticulum (calcium ion containers). The formation and maintenance of the triad is essential for muscle function^11,12^. Dysfunction of important elements, e.g. T-tubuli, in the triad is known to affect the precontractile optical response in isolated frog muscles in the lab^3–6^.

Taken together, non-invasive *in-vivo* detection and characterisation of precontractile optical response to electrostimulation in humans would be an important advance in characterising the EC process in disease. Previous *in-vivo* studies involving NIR assume spatially homogeneous processes and are limited to single-spot recordings. This may explain the lack of reported precontractile optical response in the literature.

Here, we develop a high-speed array-based NIR instrument and use a spatiotemporal approach for measuring the optical response. For the first time we non-invasively observe and characterise a precontractile spatially distributed optical signal in the electrostimulated human biceps brachii *in-vivo*. This novel finding opens up for a new category of *in-vivo* studies characterising differences in the excitation-contraction coupling in sports medicine and among patients with different neuromuscular diseases, as well as for monitoring the effects of therapy.

## Results

### Early optical response is detectable through intact skin

**Figure 1:**
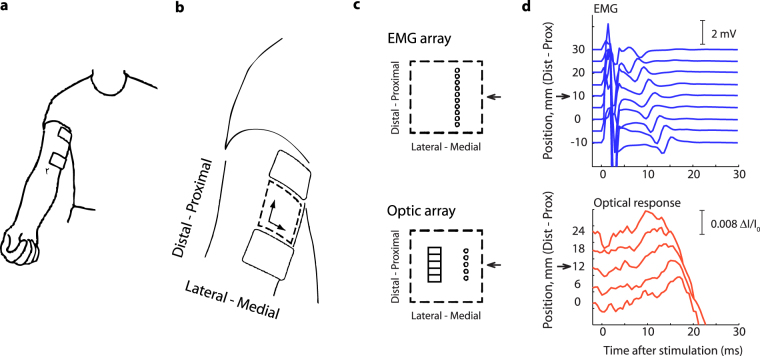
Early optical response to transcutaneous electrostimulation of the biceps brachii *in-vivo* is detectable through the intact skin. (**a**) Stimulation electrodes. (**b**) Measurement area (broken line). (**c**) Orientation of the EMG and optical probes. (**d**) Spike-triggered averages of EMG and early optical response to 25 electric stimuli in Subject 3. The traces correspond to the sensor arrays in Fig. 1c. The arrows indicate the middle sensing elements that were used for comparison of temporal characteristics.

We applied transcutaneous electrostimulation to the biceps^13^ (Fig. 1a,b) and detected optical response with a high-speed array-based NIR measurement system (Supplementary Appendix) as well as electrical response in terms of action potential (AP) with a multi channel EMG probe (Fig. 1c). Both were detected on the intact skin surface. The EMG recordings were applied because the AP initiates the excitation-contraction process and it is a well studied feature^14^ acting as a reference. The optical response and as expected the electrical response, showed waveform features in the early 0–30 ms range, similar in amplitude and shape among all channels. This was observed in all eleven subjects.

Thus optical and electrical response appeared early after electrostimulation. The early optical response was visible after a single stimulus (Supplementary Fig. 1) and was clearly visible after an average of 25 stimuli (Fig. 1d).

The waveform of the optical response over the entire contraction phase is presented in Supplementary Fig. 2. In the contractile regime (30–300 ms) the waveform characteristics were similar to those in previously reported studies^8–10^. However in the early regime (0–30 ms) the difference was distinct: where a clear peak was detected in the present study, previous *in-vivo* studies detected a “flatline”. The peak was neither an artefact from analogue nor digital filtering.

### Early optical response is precontractile and coincides with AP

**Figure 2:**
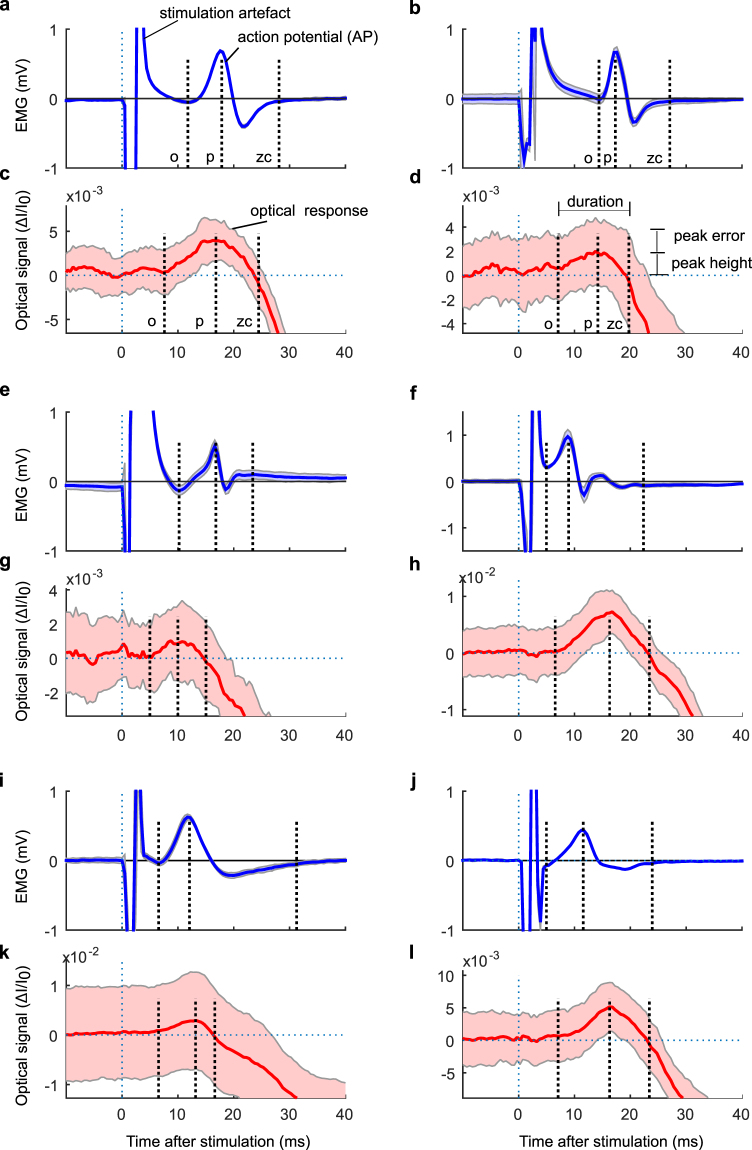
Precontractile optical response and electrical response to transcutaneous electrostimulation coincides in time. EMG and optical timing in six different subjects: (**a**,**c**) Subject 6, (**b**,**d**) Subject 3, (**e**,**g**) Subject 4, (**f**,**h**) Subject 2, (**i**,**k**) Subject 8, (**j**,**l**) Subject 10. In all subfigures O denotes onset, P denotes time to peak and ZC denotes zero-crossing of signals from the middle sensing elements (as indicated in Fig. 1). The solid lines and shaded areas indicate the means and standard deviations of 25 consecutive stimuli respectively.

The AP initiates the excitation-contraction process and therefore occurs prior to contraction^14^. In order to determine if also the optical response occurs prior to contraction we compared temporal characteristics (Fig. 2). The middle sensing element in the EMG and optical probes was selected for comparison (as indicated by the arrows in Fig. 1d). The onset (i.e. the earliest activity seen by that particular sensing element -not necessarily the global onset time in the muscle as a whole), the time to peak and the zero-crossing were identified in all eleven subjects and were found to be similar between optical and EMG responses (Table 1). Specifically, there were no significant differences in onset nor duration (Fig. 2). There was a difference in time to peak likely caused by differences in waveform, indicating the two signals carry complementary information. We consider the responses coincident in time and therefore conclude that the early optical response is actually a precontractile optical response. To clarify terminology, again, because the temporal characteristics were determined from the middle sensing element, we use the term “precontractile” not for the muscle as a whole but for the local measurement volume seen by the middle sensing element.

**Table 1:** Repeatability of temporal characteristics in early optical and EMG responses.

**Modality**	**Variable**	**S1**	**S2**	**S3**	**S4**	**S5**	**S6**	**S7**	**S8**	**S9**	**S10**	**S11**
**EMG**	Onset (O), ms	13.64 (2.99)	13.89 (−)	5.73 (0.50)	5.24 (1.19)	—	12.48 (1.83)	8.08 (0.83)	7.09 (0.30)	4.89 (0.45)	5.45 (0.41)	11.84 (0.75)
	Time-to-peak*, ms	17.07 (0.99)	17.12 (−)	9.13 (0.34)	8.35 (4.11)	—	16.96 (1.16)	11.71 (1.00)	12.15 (0.39)	8.39 (0.36)	10.01 (0.95)	16.23 (0.73)
	Duration, ms	9.04 (2.77)	11.78 (−)	10.87 (3.10)	8.54 (1.87)	—	13.27 (1.74)	13.06 (1.19)	19.95 (1.12)	11.94 (1.58)	13.31 (3.76)	13.04 (1.01)
	N	3	1	3	4	0	4	4	4	4	4	4
**NIRS**	Onset (O), ms	11.71 (1.43)	9.01 (0.98)	6.49 (0.72)	5.62 (1.14)	9.87 (3.82)	8.36 (0.89)	8.84 (0.73)	6.21 (1.91)	9.63 (2.88)	9.70 (0.25)	11.91 (0.98)
	Time-to-peak*, ms	20.97 (3.62)	15.71 (0.68)	12.15 (1.42)	9.04 (1.35)	21.11 (6.77)	15.65 (2.39)	16.65 (1.40)	12.22 (2.58)	18.06 (2.96)	16.99 (0.96)	19.45 (0.88)
	Duration, ms	16.66 (7.14)	12.86 (2.54)	10.16 (1.75)	6.46 (1.18)	18.8 (5.89)	13.0 (4.11)	16.04 (1.78)	9.22 (0.49)	12.89 (4.17)	14.33 (2.92)	15.62 (−)
	Peak height (ΔI/I_0_)	0.009 (0.004)	0.008 (0.005)	0.041 (0.017)	0.003 (0.002)	0.012 (0.003)	0.028 (0.015)	0.013 (0.008)	0.006 (0.004)	0.015 (0.009)	0.007 (0.003)	0.030 (0.021)
	Peak_error, %	66 (57)	40 (16)	18 (5)	178 (99)	28 (5)	19 (15)	42 (34)	104 (49)	21 (3)	37 (20)	23 (19)
	N	4	2	4	4	4	3	4	4	4	4	2

Mean values and (standard deviations) of the repeated measurements of EMG and NIR in Subjects 1 through 11 (S1–S11). Equality of temporal characteristics between modalities was tested using repeated measures in mixed effect models. *Marks significant differences between NIR and EMG after Bonferroni Holm correction. Poor signal quality in terms of undetectable peaks in the response was observed in some measurements yielding different numbers of repeated measurements (N). Duration was defined as the time from onset to zero-crossing. Peak error at the optical peak was defined as the standard deviation divided by the peak height (Fig. 2d).

Finally, the measurements were repeated in order to gain an idea of the repeatability/robustness of the temporal characteristics. For EMG the Intra class correlation (ICC) for the measurements were onset: 0.90, time-to-peak: 0.83 and duration: 0.66; and for NIRS; onset: 0.49, time-to-peak: 0.58 and duration: 0.45.

As expected the electrical response showed features of propagation because the AP propagates along the muscle cells. However propagation was also present in the optical response (Fig. 1d).

### Precontractile optical response propagates with the AP

To further evaluate the relationship between the precontractile optical response and the AP and to confirm that the early feature in the optical response was not an artefact, we performed spatiotemporal analysis to examine whether there was similar propagation in both responses.

**Figure 3:**
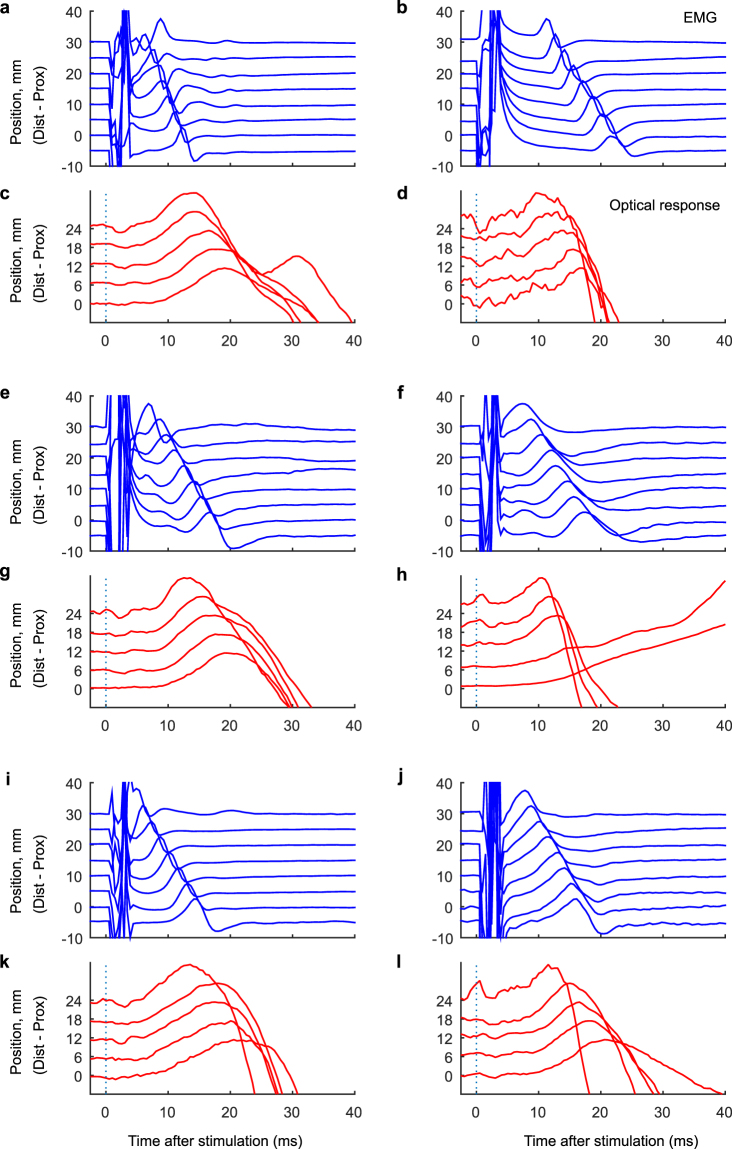
Precontractile optical response to electrostimulation propagates with the AP. EMG and optical propagation in six subjects: **(a,c)** Subject 2, **(b,d)** Subject 3, **(e,g)** Subject 7, **(f,h)** Subject 8, **(i,k)** Subject 9, **(j,l)** Subject 10. The curves are the averages of 25 consecutive stimulations. Stimulation occurred at t = 0 ms.

Propagation was found in all measurements and there were no statistical differences in absolute values of velocities. The direction of propagations was proximal to distal in eight subjects, distal to proximal in one subject and mixed (proximal to distal or distal to proximal) in two subjects. It indicates the electrostimulation was stable in all but two subjects. It also suggests that in the future, both NIR and EMG should be combined in one single probe. The conduction velocities calculated in the EMG responses were all physiologically reasonable^14^. Typical examples of optical and EMG responses are shown in Fig. 3. The repeatability of velocity was higher in the EMG response (ICC: 0.58) than in the optical response (ICC: 0.35). With the results taken together, the precontractile optical response to electrostimulation of the biceps brachii observed in this *in-vivo* study is most likely the very same as that of the isolated frog muscles observed in the *ex-vivo* studies from the previous century. At that time precontractile optical response^3,4,6^ and propagation thereof^5^ were observed in isolated muscle fibres in frogs. These early studies concluded that the optical response originates from events in the excitation-contraction process (Supplementary Fig. 2, lower part), a conclusion that is well supported by the close relationship between the optical response measured with the NIR probe and the electrical response measured with the EMG probe in the current study

Research on the EC coupling process is occurring in multiple fields. Since the 1990s there has been an explosion of information in the molecular biochemistry field^15^. Parallel to this, electromechanical delay and latency relaxation was studied from a mechanical point of view with methods such as very high frame rate ultrasound^16^ and x-ray diffraction^17^. However there are still no non-invasive methods for quantifying the time constants in the EC-coupling process.

### Inter-observer notes

Because the analysis was partly manual we let an independent observer analyse the data. The statistical conclusions for the independent observer was the same to those of the primary observer, i.e. no statistical differences in onset, duration nor propagation velocities between EMG and NIR, and a statistical difference in the time to peak.

## Discussion

Despite decades of research on NIR methods it has still not fulfilled its high level of promise and it is still a relatively blunt tool for studying muscle tissue in sports and medicine. In this *in-vivo* study, the first non-invasive observation and characterisation of a precontractile optical response to electrostimulation of skeletal muscle in humans was achieved.

A custom, high-speed, array-based NIR measurement system was applied along with transcutaneous electrostimulation of the biceps brachii muscle, and the optical response was compared to array EMG response. Temporal characteristics and velocities of propagation along the muscle cells were found to be similar in both sets of signals.

This demonstrates a precontractile optical response and suggests that that it is closely related to the excitation-contraction process i.e. the depolarisation of the cell membrane and t-tubuli system, the action of proteins and release of calcium prior to sarcomere activation^15^ and mechanical contraction (Supplementary Fig. 2, lower part).

Interestingly our results provide a significant link between the low-degrees-of-freedom *ex-vivo* studies on isolated frog muscle cells some 30–60 years ago^3–6^ and modern expectations of NIR methods. This simple method was able to detect, in humans, similar early and propagating optical response in a non-invasive many-degrees-of-freedom *in-vivo* setting, promising future opportunities for using NIR measurements in clinical evaluation of muscle tissue.

Skeletal muscle contraction is initiated by excitation from a motor neuron, triggering depolarisation of the skeletal muscle cell membrane. The excitation-contraction (EC) coupling is mediated by what is termed the triad structure within the skeletal muscle cell. This structure connects the T-tubuli system i.e. the transverse invaginations of the cell membrane, with the sarcoplasmic reticulum where calcium is stored and released onto the contractile myofilaments. Proper function of the T-tubuli system is essential to ensure synchronous calcium release throughout the cell thus providing rapid and powerful contraction. In recent years it has been found that T-tubuli structure and composition are altered in several pathological states^11^. Recent research has also revealed a class of skeletal myopathies named triadopathies^12^ where again the T-tubules are a key structure. The destruction of the T-tubuli system is known to affect precontractile optical response^4^ in isolated muscle cells. Thus, this observation of precontractile optical response is an important step towards the clinical use of non-invasive optical methods. It encourages the research community to investigate the feasibility of a non-invasive method of characterising alterations in EC coupling time and propagation characteristics by simultaneously measuring the EMG and optical response, as well as a non-invasive method for monitoring effects of therapy.

In this study the combined use of EMG and NIR not only allowed comparison of the spatiotemporal characteristics of electromyographic and optical response but also ensured the stimulated muscle tissue was actually located in the area of optical investigation. Spatiotemporal measurements of precontractile optical response may allow detection of heterogeneity in the EC coupling. The measurement depths are similar (about 10 mm) for both NIR and EMG in this setup^18,19^. The ICC for the optical response was lower than the ICC for the EMG response. This may be related to the fact the NIR probe was equipped with half the number of detectors, thus lowering the signal-to-noise ratio of the velocity estimate, and because NIR measurements are in general more sensitive for physiological differences between subjects. However the repeatability shows promise for this method to be developed into a clinical tool. In future applications, EMG and NIR spectroscopy would preferably be combined in a single probe, allowing truly simultaneous measurements. Also, in order to increase the repeatability (ICC) for the optical measurements, acceptance/rejection criteria may need to be established. One specific benefit of studying the precontractile time window is that there is no mechanical contraction and thus no gross movement artefacts corrupting measurement signals.

In this study we performed spatiotemporal investigation of optical signals 0–30 ms after electrostimulation of muscle tissue. We observed a small peak and concluded it is related to the excitation contraction process. In the later regime (30–300 ms) we observed large optical signals similar to those in previous studies^8–10^. The detailed study by Erb *et al*.^9^ concluded that the optical signals in the later regime are related to the motion of blood vessels in the measurement volume during stimulus-induced muscle contraction. All together, while the present study concludes that optical signals in the early regime are related to the excitation–contraction process, the previous studies conclude that optical signals in the later regime are related to the mechanical motion. Thus, the results complement each other.

Recently the electromechanical delay (EMD) i.e. the time lag between electrical muscle activation and muscle force production was reinvestigated with high frame rate ultrasound^16,20–23^ and MMG^24,25^. Ultrasound was used to investigate the earliest fascicle motion in the biceps brachii (3.9 ms^22^, 5.5 ms^21^, 7.1 ms^20^, 10 ms^26^). It was found that there was no difference in onset of fascicle motion and onset of myotendinous junction motion in the biceps brachii^21,23^. Thus the mechanical wave transferring fascicle to tendon motion is very fast. The velocity was measured to 30 m/s with MMG methods in the vastus lateralis muscle^24^. It was concluded that the motion of the myotendinous junction started before the AP had reached the junction and was due to the passive fascicle motion induced by contraction in the region of the motor units where the AP started^16,24^. Thus there are two known phenomena showing propagation in muscle: the AP travelling at about 4 m/s and the mechanical wave travelling at about 30 m/s. Waves in the 4 m/s region was detectable with EMG^14^, MMG^24^ and ultrasonic^16^ methods, whereas waves in the 30 m/s region was detectable with MMG^24^ methods only. In the present study we investigate the 0–30 ms time window with electrical and optical methods and detect propagation in the 4 m/s region. Although by the reasoning above there may be passive motion (>30 m/s) of muscle fascicles in the measurement volume, this seems to be undetectable with the optical method –the signals are completely dominated by a process closely coupled to the AP (4 m/s).

Two sources of error that may affect results are electrical crosstalk from the electrostimulator and optical excitation of tissue. Regarding artefacts induced by the electrostimulation, these contaminated the EMG signals somewhat and the optical signals insignificantly in the time window of investigation. In future work where the two modalities are combined in a single probe, the EMG device should be designed to be immune to the stimulation voltage. Regarding optical excitation, the optical energy estimated to 2 µJ per pulse delivered to the tissue by the optical probe is far lower than the threshold of cell membrane depolarisation reported previously^27^.

The precontractile optical response being very small and the photo detectors being very sensitive raises a crucial question: is this study based on measurements of an electric artefact from the electrostimulator? An electric artefact would 1) occur simultaneously in all PDs in the optical probe, 2) occur simultaneously with the transient stimulation, 3) occur at the same time (i.e. same time to onset) in all subjects and 4) not propagate “slowly”. Fortunately, none of these were true in the present study: The early waveform 1) did not occur simultaneously in all PDs (Fig. 3), 2) started several milliseconds after the transient stimulation (Table 1), 3) did not occur in the same time in all subjects (Table 1) and 4) propagated “slowly” (Table 2). Thus we conclude that the only electric artefact contaminating our optical signals was the single sample stimulation artefact corrected by the median filter as described in the methods section.

**Table 2:** Repeatability of propagation velocities in early EMG and optical responses.

**Modality**	**Variable**	**S1**	**S2**	**S3**	S4**	**S5**	S6***	**S7**	**S8**	S9***	**S10**	**S11**
EMG	Conduct. velocity, m/s	4.09 (0.27)	3.01 (0.45)	4.5 (0.48)	4.08 (0.69)	—	3.59 (0.21)	3.58 (0.13)	3.37 (0.18)	3.4 (0.19)	3.94 (0.47)	3.3 (0.11)
	N	3	2	3	3	0	4	4	4	4	4	4
	R^2^	0.99 (0.01)	1.00 (0.00)	0.97 (0.04)	1.00 (0.01)	—	0.99 (0.01)	1.00 (0.00)	0.99 (0.00)	0.99 (0.00)	0.99 (0.02)	1.00 (0.01)
NIRS	Veloc. of propag., m/s	4.59 (2.11)	3.76 (0.44)	4.48 (0.82)	6.58 (1.71)	2.81 (0.66)	3.57 (0.97)	2.94 (0.67)	3.81 (0.42)	4.38 (2.28)	2.96 (0.41)	3.41 (0.58)
	N	4	2	4	3	3	3	4	2	2	4	2
	R^2^	0.75 (0.225)	0.96 (0.015)	0.88 (0.137)	0.93 (0.052)	0.92 (0.038)	0.83 (0.15)	0.97 (0.025)	0.96 (0.045)	0.85 (0.084)	0.95 (0.06)	0.89 (0.138)

Mean values and (standard deviations) of the repeated measurements of EMG and NIR in Subjects 1 through 11 (S1 – S11). There were no significant differences in velocity of propagation (conduction velocity) between the responses (p = 0.07). Poor signal quality in terms of undetectable peaks in the response was observed in some measurements yielding different number of repeated measurements (N). Eight subjects had proximal to distal propagation direction, one had distal to proximal (**) and two subjects mixed directions (***).

As mentioned throughout the paper there were a few confounding factors that may have influenced the results. These were in particular 1) the manual data analysis and 2) the use of two separate probes for EMG and NIRS respectively. However 1) letting an independent observer repeat the analysis of optical signals and 2) repeating the measurements for both EMG and NIRS, we found no significant influence of these factors in the results. An additional limitation of this study is the inherent limitations in the NIRS method as implemented in this work. Because optical response was measured with a single-wavelength continuous-wave instrument absorption and scattering cannot be separated, and the specific origin of the change could not be isolated. Using more sophisticated setups, e.g. Fourier transform NIRS and hyperspectral NIRS may allow the opportunity to further investigate the exact origin of the cellular level processes of the precontractile optical response. However, the experimental setup with high sampling rate and low spatial inter-optode distance was still sufficient to resolve a spatiotemporal optical response in the same time window and with similar propogational features as the myoelectrical action potential, which has not been observed *in-vivo* before.

Further studies are necessary to develop NIR as a tool for the characterisation of alterations and heterogeneity in the EC coupling. For example, it is necessary to compare healthy subjects with subjects diagnosed with different muscular diseases. Also, a better understanding of the precise origin of precontractile optical response *in-vivo* may predict which type of muscular disease that can be diagnosed. In the meantime, our finding that precontractile optical response to electrostimulation in human muscle tissue can be detected non-invasively and *in-vivo* provides support for the use of this optical technique in medicine.

## Methods

### Subjects

Eleven healthy male subjects of age 34 (5, 28–43) years (mean (std, min-max)), height 180 (4, 173–187) cm, weight 75 (8, 66–89) kg participated in the study. Their skin and subcutaneous fat layers thicknesses were 1.2 (0.1, 1.0–1.4) mm and 1.2 (0.4, 0.6–1.7) mm respectively. They suffered from no neuromuscular disorders. The experiments were approved by The Regional Ethical Review Board in Umeå, a state-controlled organization (decision ID: 2012-300–31 M). The study was carried out in accordance with the approved guidelines. The subjects gave informed consent and were allowed to abort the experiment at any time without further explanation.

### Experimental setup and stimulation

The subject was seated comfortably in an adjustable office chair resting his arm on a table in front of him, the palm facing the midsagittal plane. The angle between the upper and lower arm was about 130 degrees. A measurement site was selected on the subject’s right upper arm (Fig. 1). Two gelled rubber electrodes for electrostimulation were carefully placed to stimulate the medial part of the biceps brachii, a procedure that was similar to Forrester and Petrofsky’s^10,13^. The electrodes were applied, one proximal to and one distal to, the measurement site. Electrical muscle stimulation was applied by a stimulator (Electrical muscle stimulator model 200, Mettler Electronics Corp, Anaheim, CA, USA). Each electrode’s active area was 2 × 2.5 cm^2^ and electrode centre-to-centre distance was about 11 cm. Transient stimulation with a pulse length of 0.5 ms and a repetition frequency of 1.5 Hz was applied. The amplitude of stimulation was increased until each pulse caused a small twitch and movement of the skin, noticeable from visual inspection, of the muscle. This low level stimulation was used in the actual recordings to minimize the number of activated motor units. High level stimulation would activate many motor units with different temporal characteristics that would superimpose and confound the detected signals thus making the comparison of temporal characteristics in EMG and optical response more complex. It would also cause larger movement of the probes causing the measurement situation to change between one stimulus and another.

### EMG recording

#### Surface EMG signals were recorded with a state-of-the-art multichannel

EMG device (Modified ActiveOne, BioSemi, Amsterdam, Netherlands). The device consists of 13 by 10 gold plated dry electrodes with 5-mm inter-electrode spacing, covering 6 by 4.5 cm of the skin’s surface (Fig. 1b, illustrated by broken line rectangle). The monopolar surface EMG signals were AD converted at 2048 Hz sample rate and 16 bit resolution.

### Optical recording

Diffuse optical signals were recorded with a custom-built NIR spectroscopy instrument based on continuous-wave near-infrared spectroscopy (Supplementary Appendix). The system allows high-resolution spatiotemporal recordings. Optimal channel separation was achieved by multiplexing the light sources and detectors. In this study, the multiplexing sequence was configured to use a single wavelength (760 nm), five light emitting diodes (LED) and five photo detectors (PD). The LEDs and PDs operated in five pairs with each pair producing one signal. The signals were sampled at 2000 Hz with 16 bit resolution. A 1,5 V trigger output from the stimulator was connected to an auxiliary input of the NIR spectroscopy instrument and to the EMG device and the trigger pulses were recorded along with the optical signals.

### Measurement procedure

**Figure 4:**
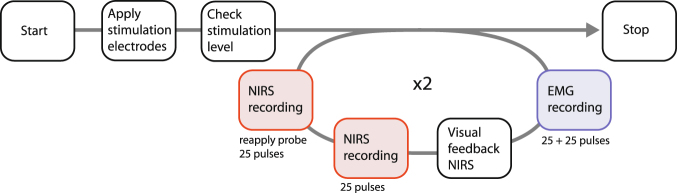
Experimental procedure.

The experiment procedure (Fig. 4) was as follows: the electro-stimulator was started up. An assistant held the EMG electrode-grid to the measurement site of the subject’s arm and 25 + 25 pulses were recorded. Then the EMG grid was removed and the assistant held the optical probe to the same measurement site. Real-time visual feedback from the NIR probe was available on a computer screen and the assistant would, within the measurement site (Fig. 1b), “go where the action was” (somewhat like an ultrasound technician looking around with an ultrasound probe) until the screen showed early features in the 0–30 ms range, similar in all five channels. Then 25 pulses were recorded. The probe was removed and reapplied and another 25 pulses were recorded. The probe was removed and the electrode grid was reapplied and another 25 + 25 pulses were recorded. Then the optical probe was applied for 25 pulses, removed, and reapplied for a last 25 pulses. The repetitions were carried out to assess the short term repeatability of the measurements.

### Analysis and statistics

The column (Fig. 1c) with the EMG signals of greatest amplitude of the EMG electrode grid was chosen for presentation of EMG signals. Analysis and statistics was performed in Matlab R2012b (Mathworks, Nattick, MA, USA) and R v3.2.3 (2015, R Core Team, Vienna, Austria). The monopolar signals were high-pass filtered at 10 Hz using a 4^th^ order Butterworth filter prior to bipolar spatial filtering to cancel common mode signals^14^. Next, stimulation event triggered averaging (a.k.a. spike-triggered averaging) was applied to calculate average and standard deviation of the EMG response.

For the optical recordings, a 2.5 ms long median filter was applied to reduce transient cross-talk stimulation artefact present in single samples during the stimulation phase. Due to the sequential sampling of the optical channels, there was a 0.5 ms delay between the samples of the first and last detector (1/2000 Hz). Simultaneously acquired signals were estimated by shifting the time signals recorded by the different detectors by their corresponding delay time using bicubic spline interpolation. In order to prevent waveform distortion causing false peaks in the optical signals no other filtering was applied.

Next the signals were divided into their individual stimulation responses. Each stimulation waveform was detrended by subtracting the baseline voltage to reduce low frequency signal drifting, calculated as the last 10 ms baseline from the previous stimulation. The final stimulation response for each of the 4 recordings was then calculated as the stimulation-event triggered averaging, similar to the EMG signals.

The relative amplitude of the optical peaks was calculated as the voltage reading of the intensity peak divided by the voltage reading of the baseline signal of each recording, ΔI/I_0_ (e.g. Fig. 2c), denoted as the Peak Height. The noise level (Peak Error) at the optical peak was defined as the standard deviation divided by the Peak Height (Fig. 2d).

The averaged EMG and optical response waveform characteristics were compared by their temporal features and propagation velocity. Temporal features (Fig. 2a,b) were manually measured by visual inspection of the waveform from the middle channels: 1) onset-time, 2) time-to-peak, and 3) time to zero-crossing after peak. Duration was subsequently calculated as time between onset and zero-crossing.

Spatial propagation was assessed by calculating the time-of-flight of the peak positions of the signals at each detector. Positions of the individual peaks were detected manually by inspection and velocity was subsequently calculated using linear regression. The R^2^ statistic from the linear regression was reported as a measure of goodness of fit.

Differences between NIR and EMG in temporal characteristics and propagation velocities were analysed using linear mixed effect models, with random effects for each subject, due to the unbalanced and repeated characteristics of the data. Intraclass Correlation (ICC) for repeatability between measurements was calculated using a random effects model. Significance level was set at 0.05.

Because a manual analysis method was used we let an independent observer (not part of our research group) manually analyse the data. The observer was a biomedical engineer working with manual and automatic data processing. Before analysis, the independent observer was shown a piece of raw data (Supplementary Fig. 1) and the entire waveform (Supplementary Fig. 2). He was then shown an EMG raw data and optical data (Fig. 1) and how the first observer would analyse these files. He was then instructed to manually measure by visual inspection (i.e. marking by mouse-clicking on a computer screen) the onset, time to peak and zero crossing in all 44 files. He was also instructed to mark the peaks in the five optical channels if there were at least three adjacent channels with similar curve form for the purpose of calculating velocity of propagation. He was not given any information about the expected outcome of the analysis.

### Data availability

The datasets generated during the current study are available from the corresponding author on reasonable request.

## Electronic supplementary material

Supplementary Figures 1 and 2 and Appendix
