# Supplementary material for: Precontractile optical response during excitation-contraction in human muscle revealed by non-invasive high-speed spatiotemporal NIR measurement
Source: Sci Rep. 2018 Jan 9;8:213. doi: 10.1038/s41598-017-18455-y (PMC5760718; doi:10.1038/s41598-017-18455-y)
Supplement: Supplementary file 1 — Supplementary Figures 1 and 2 and Appendix [file 41598_2017_18455_MOESM1_ESM.doc]

Markus Lindkvist, Gabriel Granåsen and Christer Grönlund

Precontractile optical response during excitation-contraction in human muscle revealed by non-invasive high-speed spatiotemporal NIR measurement

**Supplementary Figure 1**


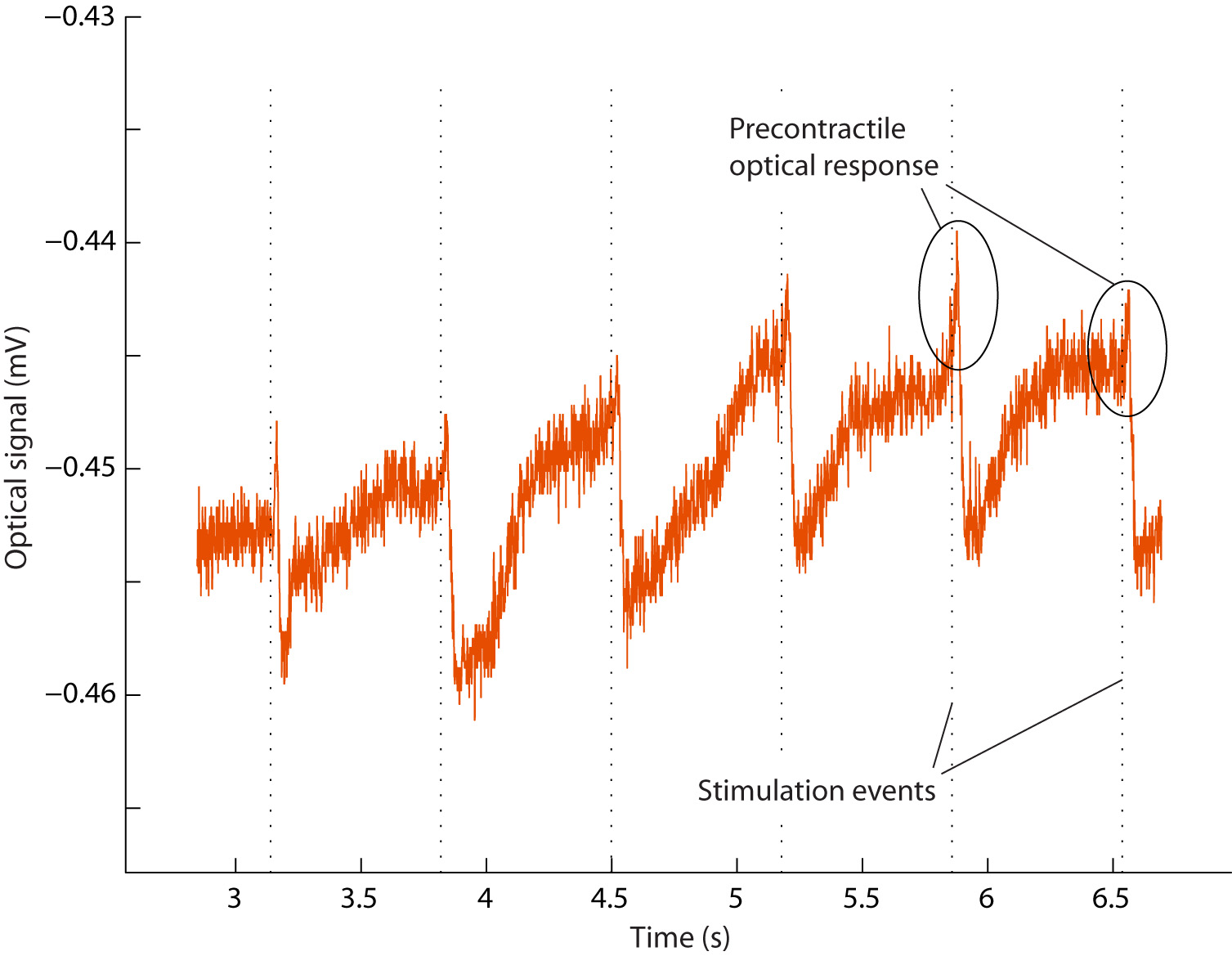


**Supplementary Figure 1 | Optical raw signal after five point medaian filtering to remove single sample stimulation artefact (subject 1).** Early optical response is visible in single stimulus.

Markus Lindkvist, Gabriel Granåsen and Christer Grönlund

Precontractile optical response during excitation-contraction in human muscle revealed by non-invasive high-speed spatiotemporal NIR measurement

**Supplementary Figure 2**


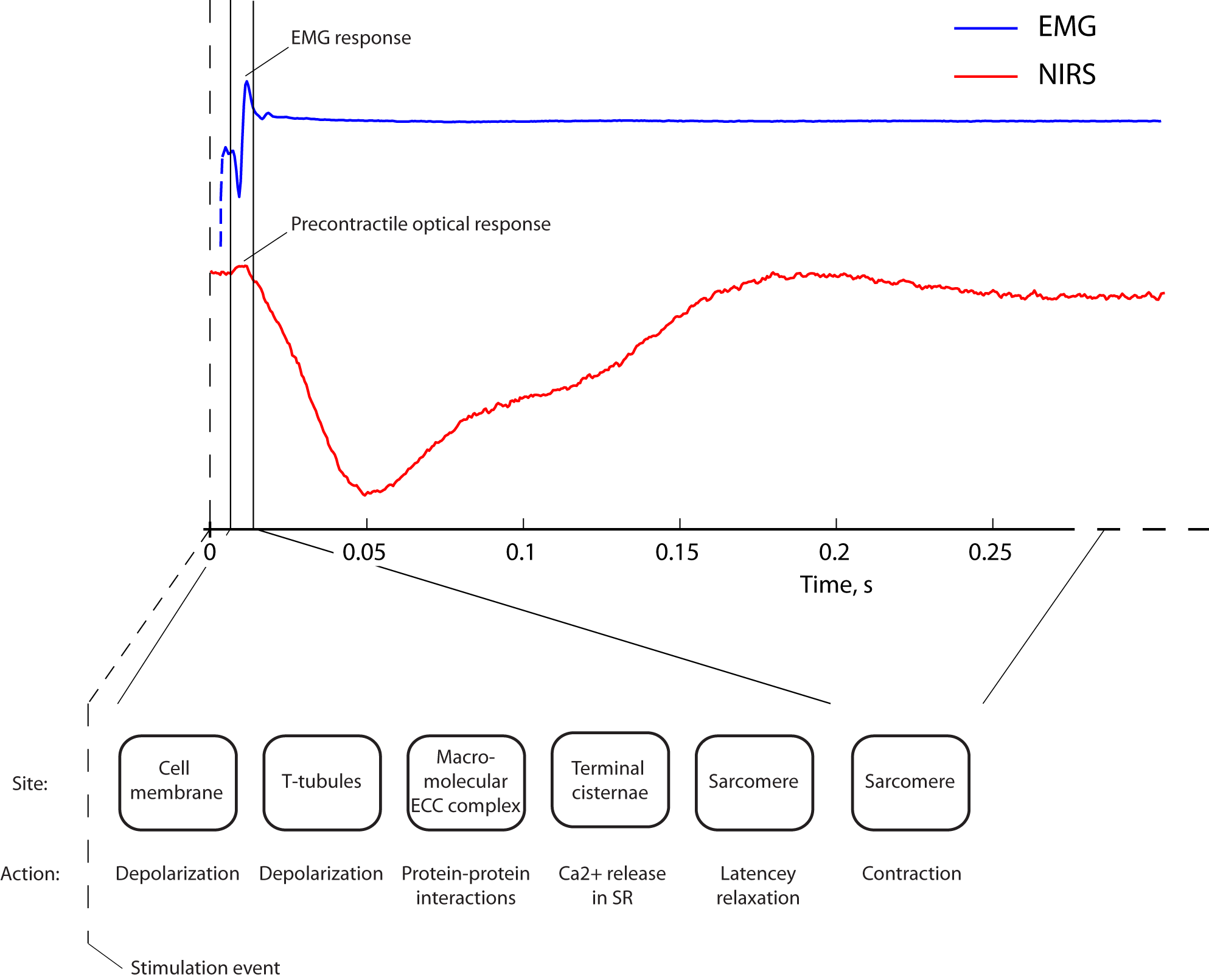


**Supplementary Figure 2 | Overview of EMG response, optical response and the chain of events in the excitation-contraction process.** (**upper**) Spike triggered average of EMG and optical response between two consecutive stimuli. The figure shows the averages of 25 consecutive stimuli from the middle channel of the probes of subject 4. In terms of visual inspection the responses in optical response are similar compared to earlier research. However there is a distinct difference in the early regime indicated by the arrow marked “Precontractile optical response”. In previous research the “bump” is missing. (**lower**) The chain of events occurring in the muscle during the excitation-contraction process. Further investigation of precontractile optical response will hopefully lead to a clinical tool for characterizing alterations in the excitation-contraction coupling mechanism in patients with neuromuscular disease and for monitoring effects of therapy.

Markus Lindkvist, Gabriel Granåsen and Christer Grönlund

Precontractile optical response during excitation-contraction in human muscle revealed by non-invasive high-speed spatiotemporal NIR measurement

**Supplementary Appendix**

# Instrumentation

This appendix describes a general-purpose modular instrument that was built for spectroscopic measurements in our research group. The instrument is a continuous wave instrument that can perform differential spectroscopy and spatially resolved spectroscopy. In this work it was used for diffuse optical measurements.

**Overview**

The instrument was built from commercially available electric components and was designed in three modules (Appendix Fig. 1): 1) A computer running a LabVIEW application (LabVIEW 2010 SP1, National instruments, Austin, TA, U.S.A.) for instrument control and data acquisition. 2) A main unit (LxWxH=300x230x110 mm3) containing a multifunction data acquisition board and patient isolation. 3) An optical probe containing light sources, photo detectors and preamplifiers.

Time multiplexing of the light sources and detectors was chosen due to its simplicity, flexibility and for optimal channel separation. The main unit is powered by a 24 VDC medical grade power supply.


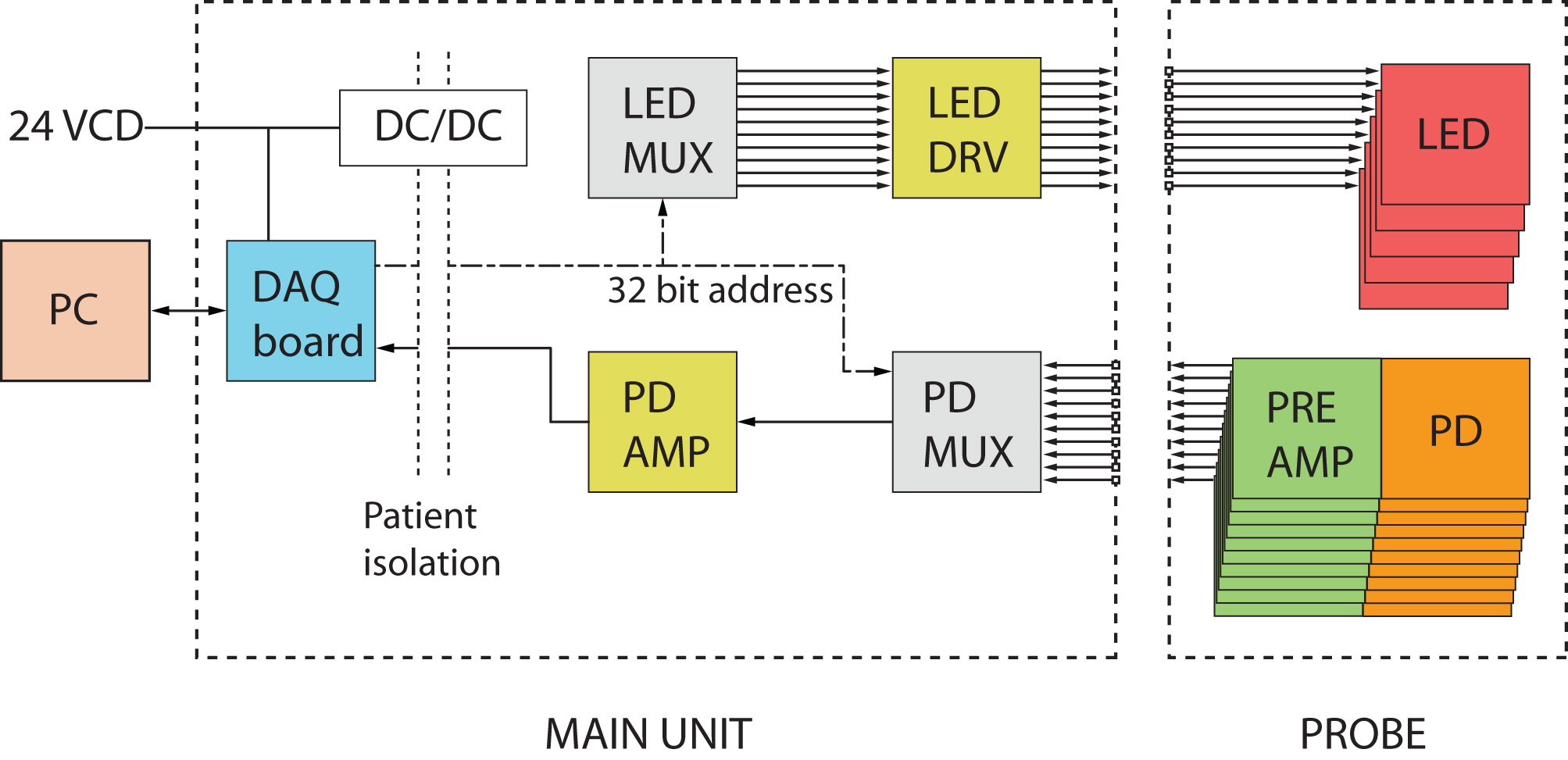


**Appendix Figure 1 | Block diagram of the NIRS system.** Personal computer (PC); isolated power supply (DC/DC); multifunction data acquisition board (DAQ board); digital multiplexer for LED control (LED MUX); driver for LED (LED DRV); light source (LED); amplifier for PD (PD AMP); demultiplexer for PD (PD MUX); preamplifier for PD (Pre AMP); photo detector (PD).

**Instrument control and data acquisition**

The software allows the user to arbitrarily set the multiplexing sequence for the probe. The multiplexing sequence is defined using a 32-bit address fed to the data acquisition board (National Instruments USB-6259) (DAQ board). The first 16 bits handle digital multiplexers (74HC4514) (LED MUX) that control light source drivers (ULN2803) (LED DRV), whereas the last 16 bits handle an analogue multiplexers (DG506) (PD MUX) that forward the analogue signals from the photo detectors (PD). The multiplexed signal is amplified and read by the data acquisition board. (A more straightforward way would have been to use the multiple analog inputs on the DAQ board but the main unit was to fit a wearable data acquisition unit 1 in a related project too.) The software demultiplexes the data, performs calculations, and presents results. Raw data can be saved to a hard drive. Because the DAQ board is placed in front of the patient isolation barrier, it can be used to record non-isolated auxiliary signals such as force, trigger, and electromyographic signals along with the NIRS signals.

**Probe**

A multi-channel array probe (Appendix Fig. 2) for diffuse optical measurements was constructed for this work. The probe consists of light sources, photo detectors, and preamplifiers (Pre AMP). The light sources were five bi-coloured light emitting diodes (Epitex L760/850-34, Ushio Epitex Inc., Kyoto, Japan) (LED). After being tested in a spectrometer, the LEDs were found to operate at 760 nm and 835 nm with spectral half-width ±25 nm and ±30 nm, respectively. The driving voltage was 5 V and the current was limited by a 3.9 Ω resistor. The photo detectors were an array detector (Hamamatsu S4114-35Q). Thirty of the detector’s 35 elements were connected in parallel three-by-three to make 10 “PDs”. Each of the 10 preamplifiers was constructed with an OP184 operational amplifier. The PD was connected to the positive and the negative inputs of the amplifier; i.e., the bias voltage was approximately zero. The feedback resistor was 300 kΩ and the bandwidth was limited to 53 kHz by a 10 pF capacitor in parallel with the feedback resistor. Because the elements of the array detector have a common cathode, the positive inputs of the op amps had to be connected to the probe ground to make a single-ended configuration. The transimpedance was 300 kΩ. All gain stages were DC-coupled and there was no light modulation to suppress ambient light. However, the flexibility of the instrument allows the user to turn off the LEDs and measure the ambient light at any place in the multiplexing sequence. The ambient light can then be subtracted from the real measurements.

In this work, in order to increase the sample rate, all of the five LEDs but only every second PD was used, only one wavelength (760 nm) was used, and there was no correction of ambient light. The effect of not correcting the ambient light was tested by switching the indoor office lights on and off. Visual inspection of the computer screen revealed no effect of the indoor lights. The lower wavelength (760 nm) was selected based on its lower absorption coefficients in water and HbO_2_ (higher depth of penetration).


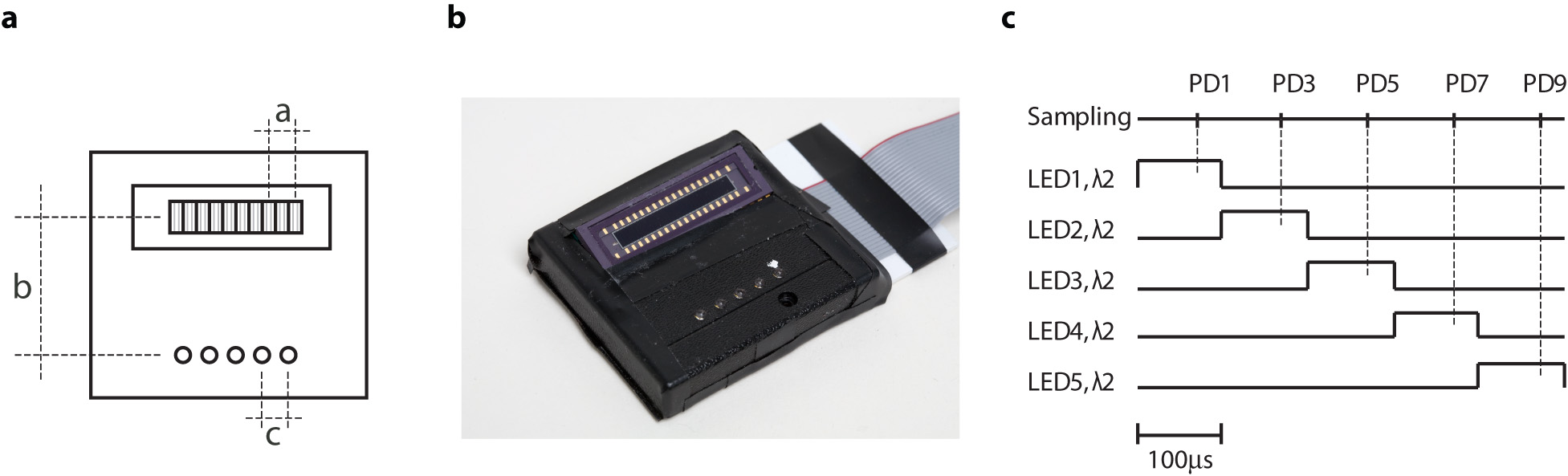


**Appendix Figure 2 |** (A) Dimensional drawing of the probe. a=6 mm, b=30 mm, c=6 mm. (B) Photograph of the probe. (C) Timing diagram for the probe. Sampling of a PD was delayed 70 µs from the initiation of the LED to allow settlement of the LED and PD circuitry.

**Sample rate**

In this instrument setup, the multiplexing sequence runs at a rate of 10 kHz, limited by the performance of the PD preamplifiers. The actual sample rate is dependent of the numbers of sources, detectors, and wavelengths. In this work the sample rate was 2 kHz. The main unit supports 24 LEDs and 24 PDs and can easily be modified to support more LEDs and PDs (hypothetically this means 216 + 216) although at the expense of sample rate.

**Patient isolation**

The patient isolation provides a galvanic isolation barrier that protects the patient against electrical hazard by separating the main parts from the applied parts. The electronics on the applied part were supplied by two DC/DC converters (THB6-2423, THB6-2411), the analogue signals were isolated with isolation amplifiers (ISO124U), and the digital signals were isolated by means of optocouplers (HCPL0631).

# References

[1] Edstrom U., Skonevik J., Backlund T. & Karlsson, J. S. A flexible measurement system for physiological signals in mobile health care. Conf. Proc. IEEE Eng. Med. Biol. Soc. 2, 2161-2 (2005).
